# Risk for development of active tuberculosis in patients with chronic airway disease—a systematic review of evidence

**DOI:** 10.1093/trstmh/trab122

**Published:** 2021-08-12

**Authors:** Yohhei Hamada, Christopher J Fong, Andrew Copas, John R Hurst, Molebogeng X Rangaka

**Affiliations:** Institute for Global Health, University College London, 30 Guilford Street, London, WC1N 1EH, UK; UCL Respiratory, University College London, London, NW3 2PF, UK; Institute for Global Health, University College London, 30 Guilford Street, London, WC1N 1EH, UK; UCL Respiratory, University College London, London, NW3 2PF, UK; Institute for Global Health, University College London, 30 Guilford Street, London, WC1N 1EH, UK; University of Cape Town, 7701 Cape Town, South Africa

**Keywords:** asthma, bronchiectasis, chronic obstructive pulmonary disease, relative risk

## Abstract

Reports suggest an increased risk of tuberculosis (TB) in people with chronic airway diseases (CADs) such as chronic obstructive pulmonary disease (COPD), but evidence has not been systematically reviewed. We performed a systematic review by searching MEDLINE and Embase for studies published from 1 January 1993 to 15 January 2021 reporting the association between the incident risk of TB in people with CADs (asthma, COPD and bronchiectasis). Two reviewers independently assessed the quality of individual studies. We included nine studies, with two from low-income high TB burden countries. Three cohort studies reported a statistically significant independent association between COPD and the risk of TB in high-income countries (n=711 389). Hazard ratios for incident TB ranged from 1.44 to 3.14 adjusted for multiple confounders including age, sex and comorbidity. There was large between-study heterogeneity (I^2^=97.0%) across studies. The direction of effect on the TB risk from asthma was inconsistent. Chronic bronchitis or bronchiectasis studies were limited. The small number of available studies demonstrated an increased risk of TB in people with COPD; however, the magnitude of the increase varies by setting and population. Data in high TB burden countries and for other CADs are limited.

## Background

Tuberculosis (TB) and chronic respiratory diseases carry a significant morbidity and mortality burden and disproportionally affect low- and middle-income countries (LMICs). Annually 10 million people develop TB and 1.5 million die.^1^ Chronic obstructive pulmonary disease (COPD), one of the most common chronic respiratory diseases, affects 251 million people and causes >3 million deaths every year. More than 90% of deaths from TB and COPD occur in LMICs.^2^

TB is an important cause of chronic respiratory disease, especially in high TB incidence countries. In a systematic review, a history of TB was associated with a 3-fold increase in the risk of COPD.^3^ A national survey in Uganda estimated that a history of TB accounted for 6% of chronic respiratory symptoms in the population.^4^ Conversely, a few studies have suggested the risk of TB in patients with COPD may be increased 2- to 3-fold.^5,6^ However, the clinical and epidemiological situation is complicated since COPD is a complex disease representing poorly reversible airflow obstruction caused by a variety of factors, especially in LMICs, which may affect its association with TB risk. Evidence suggests that smoking, the most important cause of COPD, increases the risk for development of TB.^7^ Ambient and indoor air pollution, another important cause of COPD, has also been associated with an increased risk of TB.^8,9^ Furthermore, the risk of TB may be high in individuals with COPD attributed to previous TB, who are commonly found in high TB burden countries.

Current World Health Organization (WHO) guidelines do not recommend TB preventive treatment (TPT) in people with COPD and they explicitly recommend against it in people who smoke unless they belong to other high-risk groups stipulated by the WHO, because of an unfavourable benefit–risk balance and difficulty in implementing systematic latent TB infection screening in a large population of smokers.^10^ However, treatment may be warranted in patients with COPD, who might be at a high risk for active TB.

There is less information on the association between TB and chronic respiratory diseases other than COPD with the exception of pneumoconiosis such as silicosis, which is a strong risk factor for TB.^10^ Yet there have been a few studies estimating the risk of TB in people with asthma, another type of chronic airway disease (CAD). A large population study in Singapore reported a lower risk of TB in individuals with asthma, consistent with a case–control study that reported a similar result.^11,12^ On the other hand, the use of inhaled corticosteroids in asthmatic patients may increase the risk of TB.^13^ Also, the risk of TB in people with bronchiectasis is not known, while a previous review identified studies suggesting TB as a risk factor for it.^3^

While COPD, asthma and bronchiectasis are all common CADs, no systematic review has been done to date to synthesize existing evidence on the risk of TB in patients with those diseases. The objective of this review was to investigate the risk for development of active TB in patients with CAD, including COPD, asthma and bronchiectasis. This review offers descriptive and quantitative evidence and highlights knowledge gaps that need to be filled to better inform the need for TPT in patients with CAD.

## Methods

We performed a systematic review using the Preferred Reporting Items for Systematic Reviews and Meta-analyses (PRISMA)^14^ and Meta-analysis of Observational Studies in Epidemiology (MOOSE).^15^ The protocol for this review is registered on PROSPERO (www.crd.york.ac.uk/prospero/; CRD42019136065).

### Search strategy

We searched for studies from 1 January 1993 to 15 January 2021 using MEDLINE (OVID) and Embase (OVID). We decided to search for papers published since 1993 when the Global Initiative for Asthma was established, because it was considered likely that studies published thereafter followed the standard definitions of CADs. Additionally, abstracts of the following international conferences were searched for the last 5 y: the Union World Conference on Lung Health, the American Thoracic Society Conference and the European Respiratory Society International Congress. The reference lists of included papers and review articles were also checked for additional studies. We contacted experts for additional eligible studies. No language or geographical limitations were applied. The search strategy was developed in consultation with a librarian. The detailed search strategy is presented in the appendix.

### Eligibility criteria

We included studies in individuals ≥15 y of age regardless of human immunodeficiency virus (HIV) status that reported the association between the incidence of active TB and CADs including COPD, bronchiectasis and asthma, as defined by the study authors. We also included studies reporting patients with emphysema or chronic bronchitis rather than COPD, as these terms are often used to describe a condition overlapping with COPD (but they were analysed separately in the primary analysis as described below). We included longitudinal observational cohort studies, cohorts nested within randomized or non-randomized trials and case–control studies. Case reports and case series were excluded.

The primary outcome was the incidence of bacteriologically confirmed active TB and the secondary outcome was the incidence of all TB including both bacteriologically confirmed and clinically diagnosed TB (as defined by the study authors).

### Study selection and data extraction

Two reviewers (YH and MXR) screened titles and abstracts of identified records independently then reviewed full-text articles selected through the screening process. Any discrepancies between the two reviewers were resolved through discussions.

Two investigators (YH and CF) extracted data independently using a data collection form. The following data were collected: methods (study design, total duration of the study, study context [setting, location] and date of the study). participants (number of participants, mean or median age, inclusion and exclusion criteria, smoking history, comorbidities and tuberculin skin test/interferon-γ release assay positivity), exposure (the definition of CAD and its severity and treatment) and outcomes (the number of TB cases diagnosed, methods for diagnosis of TB, definitions of clinically diagnosed TB and variables adjusted for in multivariable analyses).

### Quality of individual studies and evidence assessment

Two investigators (YH and CF) assessed the risk of bias of individual studies using the Newcastle–Ottawa Scale.^16^ The Grading of Recommendations Assessment, Development, and Evaluation methodology was used to assess and appraise the quality of evidence.^17^

### Statistical analysis and synthesis of results

The summary measures for outcomes were hazard ratio (HR), incidence rate ratio (IRR), risk ratio or odds ratio (OR), depending on the availability of data. We intended to perform meta-analyses stratified by the type of CAD and study design using fully adjusted estimates in each study. However, because we identified significant heterogeneity across studies, we do not present the meta-analysis; instead, we provide a descriptive summary of included studies and highlighted areas for further research in the discussion section. We used forest plots, I^2^ statistic and chi-squared test to measure and assess heterogeneity between included studies. We considered an I^2^ value >50% as substantial heterogeneity.

## Results

### Characteristics of included studies

**Figure 1.**
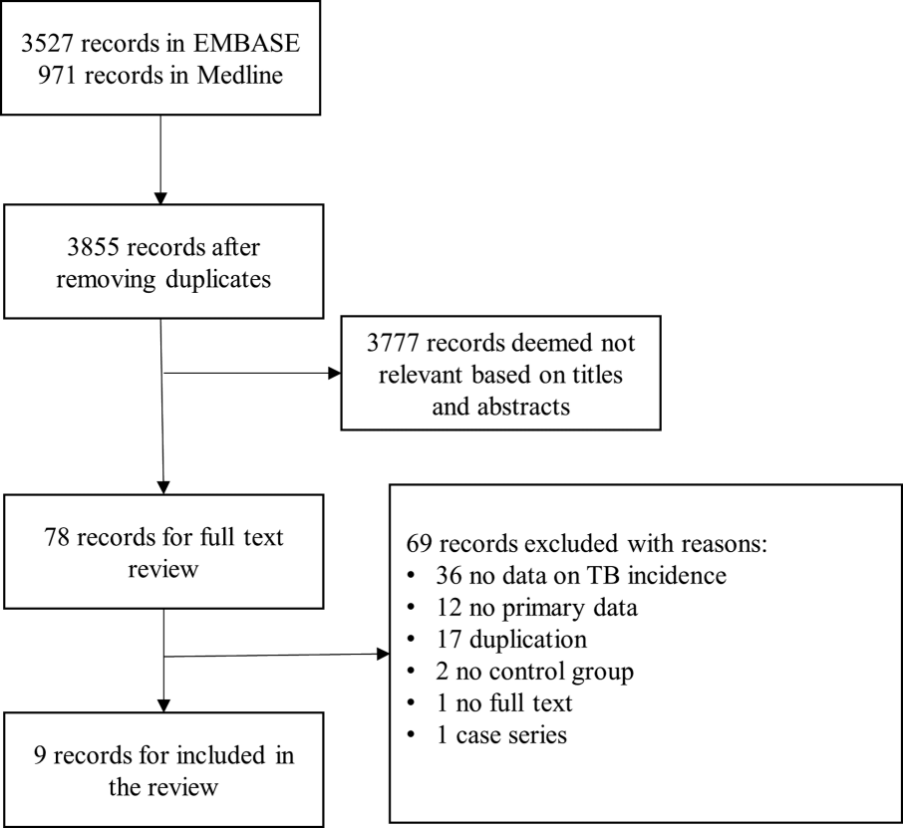
Study selection.

In total, 3855 records were identified and nine studies met our inclusion criteria (Figure 1). Seven studies were from upper-middle or high-income countries while one was conducted in India and the other was in three West African countries (Table 1).^5,6,11,12,18–22^ Four studies used data from national registries^5,6,18,22^ and reported the association between COPD and the risk of TB. Three of the four studies excluded participants with a previous history of TB.^5,6,18^ In three studies, the diagnosis of COPD was based on International Classification of Diseases, Ninth or Tenth Revision codes (ICD-9 or ICD-10, respectively),^5,6,18^ one of which additionally required prescription of COPD-specific or airway medication.^6^ An additional study reported the association between COPD and TB incidence using UK Read codes.^22^ In one of the studies including COPD patients, a nationwide study in the Republic of Korea, the primary objective was to investigate the risk of TB in patients with chronic kidney disease (CKD), with the presence or absence of COPD included as one of the covariates; hence the association between TB and COPD could be extracted only from patients with pre-dialysis CKD.^18^ Two prospective cohort studies in Singapore and the UK reported the association between incident TB and asthma while the study in Singapore also reported chronic bronchitis.^11,22^ Four studies were case–control studies; two reported the association between TB and asthma,^12,20^ one reported the association between emphysema, chronic bronchitis and asthma using the General Practice Research Database in the UK^19^ and the remaining one reported the association with bronchiectasis in patients enrolled in a hospital in Taiwan.^21^

**Table 1.** Study characteristics

Study	Design	Setting and population	Type of CAD	Definition of CAD	Diagnosis of TB and follow-up	NOS score (maximum=9)
Bhat et al., 2017^20^	Case–control	Pulmonary TB and non-TB as a control identified in a population-based survey of TB symptoms in Jabalpur, India	Asthma	Not defined	Sputum smear and/or culture-positive pulmonary TB	6
Inghammar et al., 2010^5^	Retrospective cohort	Individuals ≥40 y of age with a hospital discharge diagnosis of COPD in the nationwide inpatient register in Sweden. One control selected for each COPD case from the population register matched for sex, year of birth and county of residence	COPD	Hospital discharge diagnosis of COPD according to ICD-9 or ICD-10 codes (ICD-9: 491, 492, 496; ICD-10: J41–J44), either as a main or secondary diagnosis	Linkage with the national TB register (including both bacteriologically confirmed and clinically diagnosed). 95.1% had >1 y of follow-up	8
Jick et al., 2006^19^	Case–control	All patients with a first-time diagnosis of TB in the General Practice Research Database in the UK. Up to four controls per case matched for age, sex, the practice attended by the case and index date	Emphysema, chronic bronchitis and asthma	Based on the standardized code in the database (Read code)	Diagnosis of TB in the database with receipt of anti-TB treatment	9
Lee et al., 2013^6^	Retrospective cohort	Individuals with COPD in the national health insurance database in Taiwan. Two controls per case adjusted for age, sex and timing of entering the database	COPD	At least two visits with a COPD diagnosis according to ICD-9-CM codes (490–492, 496 and A-code A323 or A325) together with and the use of at least two COPD-specific medications (corticosteroids, β-agonists, anti-cholinergic, aminophylline and theophylline) or one COPD-specific medication plus one airway medication (oral antitussives, mucolytic agents and sympathomimetics)	Two ambulatory visits or one inpatient record with a compatible diagnosis according to ICD-9-CM, plus prescription of anti-TB treatment.Mean follow-up of 8.6 y and 8.7 y in CPD and non-CPD patients, respectively	9
Lienhardt et al., 2005^12^	Case–control	Newly detected TB patients who presented to urban health centres in The Gambia, Guiney and Guinea Bissau. Age-matched household and community control	Asthma	History and treatment of asthma collected separately. Only history of asthma was included in the multivariable regression model	Sputum smear–positive pulmonary TB	8
Park et al., 2019^18^	Retrospective cohort	Adults ≥19 y of age with pre-dialysis chronic kidney disease identified in the national health insurance database in the Republic of Korea	COPD	The presence of ICD-10 codes compatible with COPD (J41–J44) twice or more	Diagnosis of TB according to ICD-10. Median duration of follow-up 3 y	7
Ruzangi et al., 2020^22^	Retrospective cohort	All adults >18 y of age in the UK Clinical Practice Research Datalink from 1 April 2004 to 31 March 2014	COPD and asthma	Based on the standardized code in the database (Read code)	Based on UK Read codes. Follow-up from 1 month to 10 y (median 3.81 years)	6
Yii et al., 2019^11^	Prospective cohort	Ethnic Chinese adults 45–74 y of age included in a large population cohort in Singapore	Asthma and chronic bronchitis	Asthma: history of physician-diagnosed asthma. Chronic bronchitis: American Thoracic Society 1995 consensus criteria. Both ascertained through a structured interview	Linkage with the National TB Notification Registry (including both bacteriologically confirmed and clinically diagnosed). Mean duration of follow-up 17 y	8
Wu et al., 2007^21^	Case–control	Individuals with lower respiratory tract infection or who had been in contact with TB patients in a hospital in Taiwan	Bronchiectasis	Dilatation of the bronchi on high-resolution computed tomography scan	Positive culture for *Mycobacterium tuberculosis*	6

The Newcastle–Ottawa Scale scores for cohort studies ranged from 6 to 9. In two studies, cohorts with chronic respiratory diseases were not considered representative of cohorts with chronic respiratory diseases in general, as they included only hospital-discharged patients with COPD^5^ and patients with pre-CKD dialysis^18^. The study by Ruzangi et al.^22^ did not report the association between COPD and TB adjusted for covariates (Table 1 and Supplementary Table 1). In the studies by Bhat et al.^20^ and Lienhardt et al.,^12^ the ascertainment of CAD was based on interviews not blinded to case or control status.

### The risk of TB in patients with COPD

**Figure 2.**
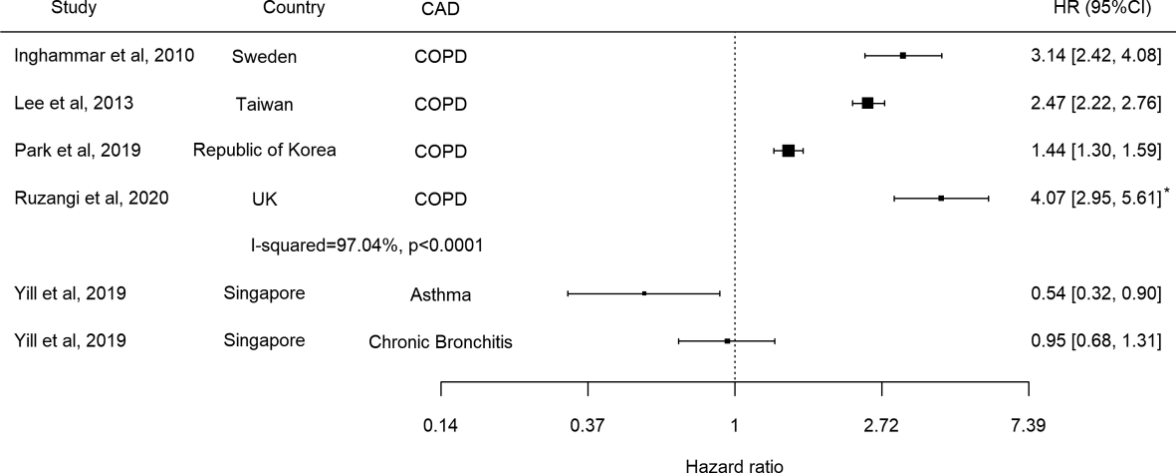
Forest plot including cohort studies. *Unadjusted IRR.

Three cohort studies reported an increase in the risk of TB in patients with COPD adjusted for multiple confounders (N=711 389) (Table 2). Adjusted HRs ranged from 1.44 to 3.14, all of which were statistically significant (Figure 2 and Table 2).^5,6,18^ The point estimate was highest in a study in Sweden including hospital-discharged COPD patients, while it was lowest in patients with pre-dialysis CKD in the Republic of Korea. There was substantial statistical heterogeneity (I^2^=97.0% [95% confidence interval {CI} 93.8 to 98.5], p<0.001), though the direction of effect was consistent. One study reported an unadjusted risk of TB in people with COPD (IRR 4.07 [95% CI 2.95 to 5.61]).^22^ While the study conducted multiple regression for the association between the risk of TB and chronic kidney diseases and included COPD as a covariate, it did not report an adjusted risk of TB in people with COPD; we contacted the authors but could not obtain data. In a prospective study by Yii et al.^11^ (N=49 762), chronic bronchitis was not associated with an increased risk of TB (HR 0.95 [95% CI 0.68 to 1.31]).

**Table 2.** Summary of findings for the association between TB and chronic pulmonary diseases

Cohort study	CAD	TB patients/participants with CAD, n/n (%)	TB patients/participants without CAD, n/n (%)	Adjusted estimate	Variables adjusted for
Inghammar et al., 2010^5^	COPD	201/115 867 (0.17)	90/115 867 (0.08)	HR 3.14 (95% CI 2.42 to 4.08)	Socio-economic status, comorbidity and immigration status, dichotomised by birth in Sweden (yes/no) and inpatient care
Lee et al., 2013^6^	COPD	674/23 594 (2.9)	554/47 188 (1.2)	HR 2.47 (95% CI 2.21 to 2.76)	Age, sex, DM, end-stage renal disease, liver cirrhosis
Park et al., 2019^18^	COPD	NA/87 427	NA/321 446	HR 1.44 (95% CI 1.30 to 1.59)	Age, sex, smoking, low income (yes or no), CKD stage, BMI, previous use of immunosuppressants, DM
Ruzangi et al., 2020^22^	COPD	NA/32 283	NA/444 929	Crude IRR 4.07 (95% CI 2.95 to 5.61)	None adjusted
	Asthma	NA/57 984	NA/419 228	Crude IRR 2.71 (95% CI 2.04 to 3.59)	
Yii et al., 2019^11^	Asthma	15/2173 (0.7)	663/47 589 (1.4)	HR 0.54 (95% CI 0.32 to 0.90)	Age, sex, dialect group, level of education
	Chronic bronchitis	46/2326 (2.0)	632/47 436 (1.3)	HR 0.95 (95% CI 0.68 to 1.31)	
Case–control study	CAD	CAD/no TB, n/n (%)	Non-CAD/control, n/n (%)	OR	Variables adjusted for
Bhat et al., 2017^20^	Asthma	68/267 (25.5)	120/1335 (8.9)	2.5 (95% CI 1.8 to 3.7)	Age, sex, occupation, annual family income, BMI, blood sugar, tobacco, alcohol consumption
Jick et al., 2006^19^	Asthma	90/497 (18.1)	185/1966 (9.4)	1.4 (95% CI 1.0 to 2.0)	Glucocorticoid use, smoking, BMI, DM, pulmonary diseases and use of anti-rheumatic or immunosuppressive agents
	Chronic bronchitis	77/497 (15.5)	154/1966 (7.8)	2.0 (95% CI1.4 to 2.9)	
	Emphysema	20/497 (4.0)	13/1966 (0.7)	3.2 (95% CI 1.3 to 7.6)	
Lienhardt et al., 2005^12^	Asthma	5/688 (0.7)	16/688 (2.3)	0.28 (95% CI 0.09 to 0.84)	Sex, HIV, smoking, marital status, family history of TB, number of adults in household, ownership of the house
Wu et al., 2007^21^	Bronchiectasis	5/264	8/438	1.3 (95% CI 0.40 to 4.2)	Age, gender, pneumoconiosis, liver cirrhosis, DM, haemodialysis, lung cancer

BMI: body mass index; DM: diabetes mellitus; NA: not available.

In a case–control study (n=2463),^19^ both chronic bronchitis (OR 2.0 [95% CI 1.4 to 2.9]) and emphysema (OR 3.2 [95% CI 1.3 to 7.6]) were associated with a significantly increased risk of TB. The quality of evidence for the association between the development of TB and COPD was considered moderate due to heterogeneity.

### The risk of TB in patients with asthma

**Figure 3.**
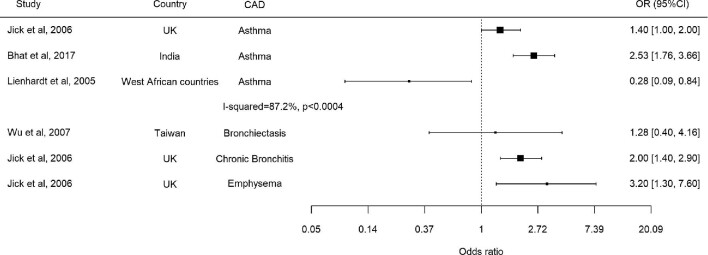
Forest plot including case–control studies.

One prospective cohort study (N=49 762) and three case–control studies (N=5441) provided data on asthma adjusted for confounders (Figure 3 and Table 2). While the cohort study reported a significantly reduced risk of TB in patients with asthma (HR 0.54 [95% CI 0.32 to 0.90]),^11^ results from the three case–control studies were inconsistent. A study in India reported a significantly increased risk of TB (OR 2.5 [95% CI 1.8 to 3.7]), while there was an inverse association in a study conducted in three West African countries.^12,20^ In a study in the UK, asthma was marginally associated with an increased risk of TB (OR 1.4 [95% CI 1.0 to 2.0]).^19^

 One study did not find a significant association between bronchiectasis and the risk of TB.

## Discussion

Our review found nine studies that investigated the risk of TB in people with CADs. Three cohort studies consistently showed a significant increase in the risk of TB in people with COPD adjusted for multiple confounders including age, sex and comorbidity. There was significant statistical heterogeneity across studies, which precluded meta-analysis. There was a lack of studies in LMICs and studies on other types of CAD were limited.

Although studies suggest a significant increase in the risk of TB in people with COPD, a causal association between COPD and the development of active TB is unclear, however, a number of hypotheses have been proposed. Impaired cellular immunity and macrophage function related to smoking in people with COPD might explain the increased risk of TB.^23^ Smoking is well known as an important risk factor for both COPD and TB.^24^ Two studies did not adjust for smoking history due to a lack of data.^5,6^ Thus the increased risk reported in those studies may be explained by smoking. However, one study in pre-dialysis patients showed a significant association after adjusting for smoking status.^18^ Also, in another study,^6^ the association between TB and COPD did not differ by sex despite the high prevalence of smoking in men according to the national statistics. These suggest that smoking alone is unlikely to fully explain the increased risk of TB observed in people with COPD. Residual confounding due to other factors such as socio-economic status, lifestyle (e.g. alcohol use) and comorbidities (diabetes and chronic kidney disease) is also possible, as they were not fully adjusted for in all studies. It is also unclear whether the increased risk of TB is due to a heightened risk for reactivation or infection. Regardless of the mechanism, it appears that individuals with COPD may be at higher risk for the development of TB, which needs further investigation.

The magnitude of the risk varied by study, ranging from 1.4-fold to 3.1-fold risk of TB. The reason for the heterogeneity is likely due to differences in study populations, settings and variables that were adjusted for. None of the studies provided data on the severity of COPD. However, the magnitude of the risk was highest in the study that included hospital-discharged COPD patients.^5^ A higher intensity of smoking exposure is suggested to be associated with a higher risk of TB, while it also increases the severity of COPD.^25,26^ Thus it is plausible that the risk of TB is higher in people with more severe COPD, but this needs further study. Development of TB in individuals with COPD causes additional lung damage and worsens lung function. The presence of COPD is reported to be associated with a higher risk of death and hospitalization from TB.^5,27^ Prevention of TB is thus important in people with COPD. TPT needs to be explored in people with COPD, particularly in those with more severe disease, taking into account the balance between its benefits and harms.

The association between asthma and TB was inconsistent. A prospective cohort study in Singapore reported an inverse association between asthma and the incidence of TB.^11^ However, as the authors discussed, adjustment for socio-economic status was not adequate. Previous studies have reported an inconsistent association between socio-economic status and the prevalence of asthma.^28^ In one study in Singapore, the prevalence of asthma in children was more common in those with higher socio-economic status.^29^ Therefore it may be possible that asthma is more common in people with higher socio-economic status who are at a lower risk of TB. Immunological mechanisms might also explain the reduction of TB risk in people with asthma. Yii et al.^11^ speculated that eosinophilic airway inflammation in asthmatic patients may protect against TB. Lienhardt et al.^12^ hypothesized that the induction of T helper type 1 (Th1) immune response by TB exposure inhibits Th2 immune response and the development of an atopic phenotype. On the other hand, the association between asthma, TB and other factors, including socio-economic status, is likely to be complex and might differ by country and setting. As an example, indoor air pollution, which is more common in poorer households, is an important risk factor for asthma in India.^30,31^ This might explain the positive association between asthma and TB reported in a study in India.^20^ Furthermore, the use of steroids in asthmatic patients may increase the risk of TB. In a study by Jick et al.,^19^ asthma remained an independent risk factor for TB after adjusting for steroid use and other variables, but residual confounding might have been possible.

Our review identified several challenges in the current literature to synthesize the evidence and inform the need for TPT. First, TB in chronic respiratory disease has received little attention over the years, as we could only identify a small number of eligible studies; this precluded investigation of heterogeneity by meta-regression and publication bias. Second, none of the included studies defined COPD according to gold standard diagnostic criteria incorporating spirometry, suggesting the presence of misclassification.^32^ Three studies that reported significant associations between COPD and TB mainly relied on ICD codes available in national databases,^5,6,18^ which might have resulted in misclassification of COPD.^33^ A study reported that the presence of more than one outpatient COPD visit based on ICD-9 had a moderate performance for identifying patients with COPD, with a sensitivity and specificity of 76% and 67%, respectively.^33^ Combining pharmacy data improves the performance but it remains imperfect.^33^ Thus the use of ICD codes could result in a large number of people incorrectly identified as having COPD. This might have led to an underestimation of the risk of TB in people with COPD. Third, most studies included clinically diagnosed TB, not just bacteriologically confirmed TB. Thus non-tuberculous mycobacterial lung disease might have been inadvertently included. This would overestimate the risk of TB. Fourth, data on the association between COPD and TB were available only from high-income countries. In LMICs with a high level of TB incidence, TB plays an important role in the development of chronic respiratory disease.^24^ A nationwide study in Uganda estimated 6% of chronic respiratory symptoms were attributed to a history of TB, a level similar to that of smoking (7%).^4^ The use of biomass fuel is another common risk factor for TB and COPD in LMICs.^34^ Thus, similar to findings from studies in high-income countries, it is likely that individuals with COPD are at higher risk of TB than those without in LMICs. However, cohort studies from these settings are needed to confirm the association. Fifth, we could not examine differences in the association by phenotype of COPD.^35^ It will be important to study which phenotypes of COPD are at the most increased risk of TB and how this could be modulated by treatment of COPD.

## Conclusions

The small number of studies suggests that people with COPD are at an increased risk of TB and the magnitude of the risk is likely to vary by setting and population. We need more studies on the risk of TB in people with CADs diagnosed using the standard criteria to identify those who are at the highest risk of TB, particularly in LMICs.

## Supplementary Material

trab122_Supplemental_File

## Data Availability

The data underlying this article are available in the article and in its online supplementary material.

## References

[1] World Health Organization . Global tuberculosis report 2020. Geneva: World Health Organization.

[2] World Health Organization . Chronic obstructive pulmonary disease (COPD). Available from: https://www.who.int/news-room/fact-sheets/detail/chronic-obstructive-pulmonary-disease-(copd) [accessed 19 December 2020].

[3] Abouda M , Yangui F, Triki M et al. Prévention de la tuberculose. Rev Pneumol Clin. 2015;71(2–3):159–67. 25282572 10.1016/j.pneumo.2014.06.002

[4] van Kampen SC , Jones R, Kisembo H et al. Chronic respiratory symptoms and lung abnormalities among people with a history of tuberculosis in Uganda: a national survey. Clin Infect Dis. 2019;68(11):1919–25. 30239605 10.1093/cid/ciy795

[5] Inghammar M , Ekbom A, Engstrom G et al. COPD and the risk of tuberculosis—a population-based cohort study. PLoS One. 2010;5(4):e10138. 20405056 10.1371/journal.pone.0010138 PMC2854124

[6] Lee CH , Lee MC, Shu CC et al. Risk factors for pulmonary tuberculosis in patients with chronic obstructive airway disease in Taiwan: a nationwide cohort study. BMC Infect Dis. 2013;13:194. 23631563 10.1186/1471-2334-13-194 PMC3652752

[7] Slama K , Chiang CY, Enarson DA et al. Tobacco and tuberculosis: a qualitative systematic review and meta-analysis. Int J Tuberc Lung Dis. 2007;11(10):1049–61. 17945060

[8] Obore N , Kawuki J, Guan J et al. Association between indoor air pollution, tobacco smoke and tuberculosis: an updated systematic review and meta-analysis. Public Health. 2020;187:24–35. 32889229 10.1016/j.puhe.2020.07.031

[9] Kim H , Yu S, Choi H. Effects of particulate air pollution on tuberculosis development in seven major cities of Korea from 2010 to 2016: methodological considerations involving long-term exposure and time lag. Epidemiol Health. 2020;42:e2020012. 32164052 10.4178/epih.e2020012 PMC7285441

[10] World Health Organization . WHO consolidated guidelines on tuberculosis: module 1: prevention: tuberculosis preventive treatment. Geneva: World Health Organization, 2020. 32186832

[11] Yii AC , Soh AZ, Chee CBE et al. Asthma, sinonasal disease, and the risk of active tuberculosis. J Allergy Clin Immunol Pract. 2019;7(2):641–8.e1. 30130591 10.1016/j.jaip.2018.07.036 PMC6363891

[12] Lienhardt C , Fielding K, Sillah JS et al. Investigation of the risk factors for tuberculosis: a case-control study in three countries in West Africa. Int J Epidemiol. 2005;34(4):914–23. 15914505 10.1093/ije/dyi100

[13] Lee C-H , Kim K, Hyun MK et al. Use of inhaled corticosteroids and the risk of tuberculosis. Thorax. 2013;68(12):1105–13. 23749841 10.1136/thoraxjnl-2012-203175

[14] Liberati A , Altman DG, Tetzlaff J et al. The PRISMA statement for reporting systematic reviews and meta-analyses of studies that evaluate healthcare interventions: explanation and elaboration. BMJ. 2009;339:b2700. 19622552 10.1136/bmj.b2700 PMC2714672

[15] Stroup DF , Berlin JA, Morton SC et al. Meta-analysis of observational studies in epidemiology: a proposal for reporting. Meta-analysis Of Observational Studies in Epidemiology (MOOSE) group. JAMA. 2000;283(15):2008–12. 10789670 10.1001/jama.283.15.2008

[16] Wells G , Shea B, O'Connell D et al. The Newcastle–Ottawa Scale (NOS) for assessing the quality of non-randomized studies in meta-analysis. Available from: http://www.ohri.ca/programs/clinical_epidemiology/oxford.asp[accessed 1 August 2021].

[17] Guyatt G , Oxman AD, Akl EA et al. GRADE guidelines: 1. Introduction–GRADE evidence profiles and summary of findings tables. J Clin Epidemiol. 2011;64(4):383–94. 21195583 10.1016/j.jclinepi.2010.04.026

[18] Park S , Lee S, Kim Y et al. Association of CKD with incident tuberculosis. Clin J Am Soc Nephrol. 2019;14(7):1002–10. 31171591 10.2215/CJN.14471218 PMC6625615

[19] Jick SS , Lieberman ES, Rahman MU et al. Glucocorticoid use, other associated factors, and the risk of tuberculosis. Arthritis Rheum. 2006;55(1):19–26. 16463407 10.1002/art.21705

[20] Bhat J , Rao VG, Sharma RK et al. Investigation of the risk factors for pulmonary tuberculosis: a case–control study among *Saharia* tribe in Gwalior district, Madhya Pradesh, India. Indian J Med Res. 2017;146(1):97–104. 29168465 10.4103/ijmr.IJMR_1029_16 PMC5719614

[21] Wu HP , Pan YH, Hua CC et al. Pneumoconiosis and liver cirrhosis are not risk factors for tuberculosis in patients with pulmonary infection. Respirology. 2007;12(3):416–9. 17539848 10.1111/j.1440-1843.2007.01033.x

[22] Ruzangi J , Iwagami M, Smeeth L et al. The association between chronic kidney disease and tuberculosis; a comparative cohort study in England. BMC Nephrol. 2020;21:420. 32998703 10.1186/s12882-020-02065-4 PMC7528250

[23] O'Toole RF , Shukla SD, Walters EH. TB meets COPD: an emerging global co-morbidity in human lung disease. Tuberculosis (Edinb). 2015;95(6):659–63. 26386744 10.1016/j.tube.2015.08.005

[24] Sarkar M , Srinivasa Madabhavi I et al. Tuberculosis associated chronic obstructive pulmonary disease. Clin Respir J. 2017;11(3):285–95. 28268242 10.1111/crj.12621

[25] Lin HH , Ezzati M, Murray M. Tobacco smoke, indoor air pollution and tuberculosis: a systematic review and meta-analysis. PLoS Med. 2007;4(1):e20. 17227135 10.1371/journal.pmed.0040020 PMC1769410

[26] Forey BA , Thornton AJ, Lee PN. Systematic review with meta-analysis of the epidemiological evidence relating smoking to COPD, chronic bronchitis and emphysema. BMC Pulm Med. 2011;11:36. 21672193 10.1186/1471-2466-11-36 PMC3128042

[27] Attia EF , McGinnis KA, Feemster LC et al. Association of COPD with risk for pulmonary infections requiring hospitalization in HIV-infected veterans. J Acquir Immune Defic Syndr. 2015;70(3):280–8. 26181820 10.1097/QAI.0000000000000751 PMC4607625

[28] Hancox RJ , Milne BJ, Taylor DR et al. Relationship between socioeconomic status and asthma: a longitudinal cohort study. Thorax. 2004;59(5):376–80. 15115861 10.1136/thx.2003.010363 PMC1747001

[29] Goh DY , Chew FT, Quek SC et al. Prevalence and severity of asthma, rhinitis, and eczema in Singapore schoolchildren. Arch Dis Child. 1996;74(2):131–5. 8660075 10.1136/adc.74.2.131 PMC1511500

[30] Singh SK , Gupta J, Sharma H et al. Socio-economic correlates and spatial heterogeneity in the prevalence of asthma among young women in India. BMC Pulm Med. 2020;20(1):190. 32664897 10.1186/s12890-020-1124-z PMC7362630

[31] Jindal SK , Aggarwal AN, Jindal A. Household air pollution in India and respiratory diseases: current status and future directions. Curr Opin Pulm Med. 2020;26(2):128–34. 31724964 10.1097/MCP.0000000000000642

[32] The Global Initiative for Chronic Obstructive Lung Disease . Global strategy for the diagnosis, management and prevention of chronic obstructive pulmonary disease (2020 report). Available from: https://goldcopd.org/wp-content/uploads/2019/12/GOLD-2020-FINAL-ver1.2-03Dec19_WMV.pdf [accessed 1 August 2021].

[33] Cooke CR , Joo MJ, Anderson SM et al. The validity of using ICD-9 codes and pharmacy records to identify patients with chronic obstructive pulmonary disease. BMC Health Serv Res. 2011;11:37. 21324188 10.1186/1472-6963-11-37 PMC3050695

[34] van Gemert F , Chavannes N, Kirenga B et al. Socio-economic factors, gender and smoking as determinants of COPD in a low-income country of sub-Saharan Africa: FRESH AIR Uganda. NPJ Prim Care Respir Med. 2016;26:16050. 27597659 10.1038/npjpcrm.2016.50 PMC5011937

[35] Miravitlles M , Soler-Cataluña JJ, Calle M et al. Treatment of COPD by clinical phenotypes: putting old evidence into clinical practice. Eur Respir J. 2013;41(6):1252–6. 23060631 10.1183/09031936.00118912

