# Supplementary material for: Risk for development of active tuberculosis in patients with chronic airway disease—a systematic review of evidence
Source: Trans R Soc Trop Med Hyg. 2021 Aug 12;116(5):390–8. doi: 10.1093/trstmh/trab122 (PMC9070518; doi:10.1093/trstmh/trab122)
Supplement: trab122_Supplemental_File [file trab122_supplemental_file.docx]

Appendix.

**Search strategy**

Medline

| Chronic respiratory disease |  | Lung Diseases, Obstructive/ |
| --- | --- | --- |
|  |  | exp Pulmonary Disease, Chronic Obstructive/ |
|  |  | emphysema$.ti,ab,kf. |
|  |  | (chronic$ adj3 bronchiti$).ti,ab,kf. |
|  |  | (obstruct$ adj3 (pulmonary or lung$ or airway$ or airflow$ or bronch$ or respirat$)).ti,ab,kf. |
|  |  | COPD.ti,ab,kf. |
|  |  | COAD.ti,ab,kf. |
|  |  | COBD.ti,ab,kf. |
|  |  | AECB.ti,ab,kf. |
|  |  | Or/1-9 |
|  |  | exp asthma/ |
|  |  | asthma$.ti,ab,kf. |
|  |  | Or/11-12 |
|  |  | (bronchopulmonar$ adj3 aspergillosis).ti,ab,kf. |
|  |  | exp Bronchiectasis/ |
|  |  | bronchiect$.ti,ab,kf. |
|  |  | bronchoect$.ti,ab,kf. |
|  |  | kartagener$.ti,ab,kf. |
|  |  | (ciliary adj3 dyskinesia).ti,ab,kf. |
|  |  | (bronchial$ adj3 dilat$).ti,ab,kf. |
|  |  | or/ 10,13, 14-20 |
| Tuberculosis |  | exp tuberculosis/ OR tubercul*.ti,ab,kf. |
| Study design |  | exp cohort studies/ |
|  |  | cohort$.tw. |
|  |  | controlled clinical trial.pt. |
|  |  | epidemiologic methods/ |
|  |  | exp case-control studies/ |
|  |  | (case$ and control$).tw. |
|  |  | longitudinal studies.ti,ab,kf. OR longitudinal study.ti,ab,kf. |
|  |  | prospective studies.ti,ab,kf. OR prospective study.ti,ab,kf. |
|  |  | retrospective study.ti,ab,kf. OR retrospective studies.ti,ab,kf. |
|  |  | follow-up study.ti,ab,kf. OR follow-up studies.ti,ab,kf. |
|  |  | observational study.ti,ab,kf. OR observational studies.ti,ab,kf. |
|  |  | or/23-33 |
|  |  | and/21,22, 34 |
|  |  | case reports.pt. |
|  |  | 35 not 36 |
|  |  | limit 37 to yr="1993 -Current" |

EMBASE

| Chronic respiratory disease |  | Lung Diseases, Obstructive/ |
| --- | --- | --- |
|  |  | Chronic Obstructive Lung Disease/ |
|  |  | Obstructive Airway Disease/ |
|  |  | Chronic Bronchitis/ |
|  |  | Lung Emphysema/ |
|  |  | emphysema$.ti,ab,kw. |
|  |  | (chronic$ adj3 bronchiti$).ti,ab,kw. |
|  |  | (obstruct$ adj3 (pulmonary or lung$ or airway$ or airflow$ or bronch$ or respirat$)).ti,ab,kw. |
|  |  | COPD.ti,ab,kw. |
|  |  | COAD.ti,ab,kw. |
|  |  | COBD.ti,ab,kw. |
|  |  | AECB.ti,ab,kw. |
|  |  | Or/1-12 |
|  |  | exp asthma/ |
|  |  | asthma$.ti,ab,kw. |
|  |  | Or/14-15 |
|  |  | exp Bronchiectasis/ |
|  |  | bronchiect$.ti,ab,kw. |
|  |  | bronchoect$.ti,ab,kw. |
|  |  | kartagener$.ti,ab,kw. |
|  |  | (ciliary adj3 dyskinesia).ti,ab,kw. |
|  |  | (bronchial$ adj3 dilat$).ti,ab,kw. |
|  |  | or/13,16,17-22 |
| Tuberculosis |  | tuberculosis/ OR latent tuberculosis/ OR post primary tuberculosis/ OR primary tuberculosis/ OR lung tuberculosis/ OR (tuberculosis OR tuberculoses).ti,ab,kw. |
| Study design |  | exp cohort analysis/ |
|  |  | cohort$.ti,ab,kw. |
|  |  | exp epidemiology/ |
|  |  | exp longitudinal study/ |
|  |  | exp prospective study/ |
|  |  | exp follow up/ |
|  |  | exp case control study/ |
|  |  | (case$ and control$).tw. |
|  |  | longitudinal studies.ti,ab,kw. OR longitudinal study.ti,ab,kw. |
|  |  | prospective studies.ti,ab,kw. OR prospective study.ti,ab,kw. |
|  |  | retrospective study.ti,ab,kw. OR retrospective studies.ti,ab,kw. |
|  |  | follow-up study.ti,ab,kw. OR follow-up studies.ti,ab,kw. |
|  |  | observational study.ti,ab,kw. OR observational studies.ti,ab,kw. |
|  |  | or/25-37 |
|  |  | and/23, 24, 38 |
|  |  | case report/ OR editorial/ |
|  |  | 39 not 40 |
|  |  | limit 41 to yr="1993 -Current" |
|  |  | limit 42 to (conference abstracts or embase) |

**Supplementary table.** Risk of bias assessment using the Newcastle-Ottawa Scale.

| Cohort study | | | | | | | | | |
| --- | --- | --- | --- | --- | --- | --- | --- | --- | --- |
| Reference | Representativeness of exposed cohort | Selection of non-exposed cohort | Ascertainment of exposure | Outcome not present at start of study | Comparability | Assessment of outcomes | Length of follow-up | Adequacy of follow-ups | Total |
| Inghammar et al, 2010 | Hospital-discharged patients with COPD | * | * | * | ** | * | * | * | 8 |
| Lee et al, 2013 | * | * | * | * | ** | * | * | * | 9 |
| Park et al, 2019 | Only patients with pre-dialysis CKD | * | * |  | ** | * | * | * | 7 |
| Ruzangi et al, 2020 | * | * | * | * |  | * |  | * | 6 |
| Yii et al, 2019 | * | * | * |  | ** | * | * | * | 8 |
| Case-control study | | | | | | | | | |
|  | Case definition | Representativeness of cases | Selection of controls | Definition of controls | Comparability | Ascertainment of exposure | The same method of ascertainment | non-response rate | Total |
| Bhat, et al 2017 | * | Only symptomatic TB | * | * | ** |  | * |  | 6 |
| Jick et al, 2006 | * | * | * | * | ** | * | * | * | 9 |
| Lienhardt, et al 2005 | * | * | * | * | ** |  | * | * | 8 |
| Wu et al 2007 | * | Only patients who present to a single hospital with lower respiratory tract infection or who  had been in contact with TB patients | Hospital control | * | ** | * |  | * | 6 |

COPD: chronic obstructive pulmonary disease; TB: tuberculosis; CKD: chronic kidney disease
